# Penetration of Gastric Ulcer into the Splenic Artery: A Rare Complication

**DOI:** 10.4021/gr2009.10.1321

**Published:** 2009-11-20

**Authors:** Lakshmi Pasumarthy, Rahul R. Kumar, James Srour, Duane Ahlbrandt

**Affiliations:** a York Hospital, 1001 S. George Street, York, PA 17405, USA

**Keywords:** Gastric ulcer, Splenic artery, Penetration, Arteriogram

## Abstract

Gastric ulcer and its complications are commonly encountered by physicians. Penetration is only noted in about 20% of the cases. Penetration into the splenic artery is a very rare occurrence. Making the diagnosis early on is important in order to prevent morbidity and mortality due to the brisk bleeding that can follow. In our report we describe the case, the imaging and the interventional radiological findings which helped with management. We review the literature published including radiological findings in such cases and describe the proposed pathogenesis. Definitive treatment usually involves controlling the bleeding either by means of embolization using coils or ligating the bleeding vessel.

## Case Report

A 55-year-old white male presented to emergency room with pain in the abdomen, hematemesis, and melena. The abdominal pain was ongoing for 2 months, in the left upper quadrant, at times worsened with eating, and had abruptly increased in severity on the morning of admission associated with a few bouts of hemetemesis and melena. He denied any chest pain or shortness of breath.

His past medical history was significant for hypertension, hyperlipidemia, diabetes mellitus and coronary artery disease. He was on metformin, aspirin, simvastatin and metoprolol. He denied any other NSAID use. He had a thirty pack year history of smoking, drank alcohol occasionally and denied any illicit drug use.

On examination his temperature was 97degrees, heart rate was 102/ minute, sinus tachycardia, blood pressure was 135/85 mmHg, respiratory rate 13/ minute, oxygen saturation was 100% on two liters of oxygen. He was pale and uncomfortable. His abdomen was soft, tender in the left upper quadrant with bowel positive sounds. Rectal examination was grossly heme-positive. The rest of the physical examination was within normal limits.

Laboratory results showed white cell count of 11.4 K/mcL, hemoglobin 8.1 gm/dl, hematocrit 25 %, platelet count 372 K/mcL, normal renal panel, blood urea nitrogen 14 mg/dl, creatinine 1.1 mg/dl, glucose 231 mg/dl and lactic acid level was 7 mmol/L. Prothrombin time was 11.9 seconds, International Normalized Ratio was 1.1 and Partial thromboplastin time was 27.6 seconds. Computed tomography (CT) scan of abdomen at that time showed a small 2.5 x 2.6 cm infrarenal aortic aneurysm and 2.1 cm right common iliac artery aortic aneurysm.

**Figure 1:**
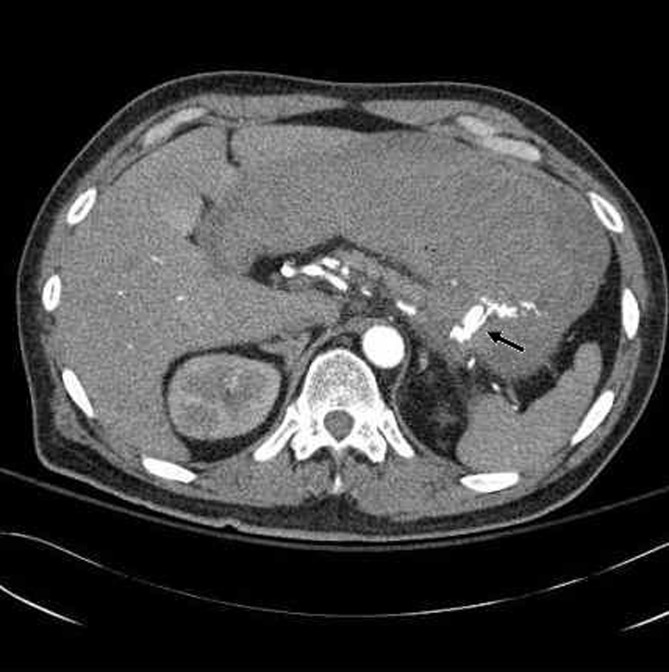
Intravenous contrast extravasating into the stomach from the splenic artery.

**Figure 2:**
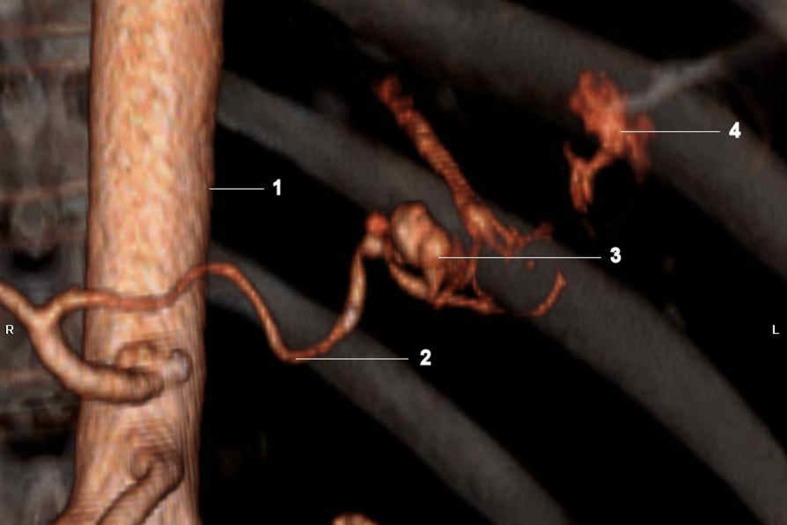
(1) Abdominal aorta; (2) Splenic artery; (3) Aneurysm of splenic artery- incidental;(4) Active extravasation of contrast from splenic artery where the gastric ulcer was eroding into it.

He became hemodynamically unstable later with blood pressure of 79/ 39mm Hg, heart rate of 140/ minute. Emergent CT scan with contrast showed active contrast extravasation into the stomach from the splenic artery, raising the possibility of penetrating gastric ulcer involving these vessels (Fig. 1). Following that, he underwent emergent splenic arteriogram which confirmed an area of active contrast extravasation from the mid portion of the splenic artery beyond the dorsal pancreatic artery (Fig. 2). The splenic artery was embolized with 6 mm x 14 cm fibered micro coils both proximal and distal to the site of extravasation. Follow-up splenic and celiac arteriogram showed flow in the splenic artery to the level of the dorsal pancreatic but not distal. He did well over the next day but the left sided abdominal pain returned. Repeat contrast enhanced CT scan showed continued extravasation of blood into the stomach and he was taken to operating room. The stomach had a very large ulcer which was penetrating into the pancreas. The top of this ulcer was only 2 cm away from the esophagus, and therefore to perform resection of the ulcer, a total gastrectomy had to be performed. Splenectomy was also performed since the spleen was found to be infarcted.

Histopathology of the spleen revealed evidence of hemorrhagic infarction without evidence of vasculitis or vascular dysplasia identified. The gastric ulcer was 5.4 x 3.7 cm, with granulation tissue, gastric mucosa with fresh hemorrhage and reactive changes. No Helicobacter pylori organisms were identified. No malignancy was identified.

The patient recuperated and was later on discharged home.

## Discussion

The two main risk factors implicated in etiopathogenesis of peptic ulcers are Helicobacter pylori and NSAIDS. The most common complications of gastric ulcers are bleeding, perforation, penetration and gastric outlet obstruction. The most common organ involved in penetration by an ulcer is the pancreas, owing to the proximity of stomach and duodenum to the pancreas.

Since 1989, approximately 500,000 people have been diagnosed with gastric ulcer but the prevalence is declining owing to the declining prevalence of H. pylori. However, the incidence of potentially life-threatening ulcer complications has not declined in spite of advances made in the management of peptic ulcer disease, secondary to increase in NSAID use, especially the use of aspirin for cardio-protective purposes [1]. Epidemiologic studies indicate that the elderly are more prone to these complications [2]. Giant ulcers (more than 3 cm in diameter) may be associated with a higher rate of complications [3]. Penetration by the ulcer refers to erosion of ulcer through the bowel wall without free perforation and leakage of luminal contents into the peritoneal cavity. Penetration often comes to attention because of a change in symptoms usually involving a loss of association of the pain with meals, and loss of relief with food and medication. The commonest sites of secondary involvement are the pancreas, gastrohepatic omentum, liver, biliary tract, colon and mesocolon [4].

Usually when a gastric ulcer penetrates into blood vessels it involves the gastroduodenal and left gastric arteries [5]. Only a few reports have mentioned penetration of gastric ulcer into splenic artery [6, 7] and in almost all the reported cases the massive bleeding is preceeded by sentinel or herald bleeds. This rare phenomenon may just be due the fact that the hemorrhage is catastrophic and sometimes diagnosis is made post mortem. Moreover, the artery is anatomically separated from the posterior wall of the stomach by the pancreas, into which the ulcer must penetrate quite deeply. Therefore radiologic signs of ulcer penetrating into pancreas must be recognised.

The most convenient, widely utilized radiologic technique to confirm diagnosis in the case of penetration is a contrast enhanced CT scan of abdomen. Madrazo et al reported preoperative diagnosis utilizing CT examination in four cases of pancreatic penetration by peptic ulcers [8]. Only one examination, however, revealed an ulcer crater extending into the pancreas. Findings common to three of the four cases included an extra-alimentary ulcer crater, a sinus tract between the ulcer crater and the head of the pancreas, and enlargement of the head of the pancreas. Specific radiologic signs of penetration were also described as loss of fascial planes between the stomach and pancreas and bands of soft-tissue density connecting these structures [9]. There are reports of erosion into splenic artery by malignancies.

The management includes hemodynamically stabilising the patient, discontinuing NSAIDS if implicated. Surgical intervention is always necessary and should be instituted expeditiously in order to minimise adverse events from blood loss. In all cases including ours, this involves either partial or sub-total gastrectomy, or proximal and distal ligation (embolization in our case).

In summary, splenic artery erosion is rare result of gastric ulcer penetration into the pancreas. Surgical intervention should be prompt to ensure a positive outcome.

### Learning points

(1) Giant gastric ulcers are more likely to cause complications such as penetration; (2) Penetration often comes to attention because of a change in symptoms; (3) Usually when a gastric ulcer penetrates into blood vessels, it involves the gastroduodenal and left gastric arteries.
